# Community-engaged clinical governance and machine learning for optimizing tuberculosis management in rural Eastern Cape

**DOI:** 10.3389/fpubh.2025.1713401

**Published:** 2026-02-13

**Authors:** Lindiwe Modest Faye, Ntandazo Dlatu, Mojisola Clara Hosu, Wezile Wilson Chitha, Teke Apalata

**Affiliations:** 1 School of Laboratory Medicine and Pathology, Faculty of Medicine and Health Sciences, Walter Sisulu University, Mthatha, South Africa; 2 Walter Sisulu Institute for Clinical Governance, Healthcare Administration, School of Public Health, Faculty of Medicine and Health Sciences, Walter Sisulu University, Mthatha, South Africa

**Keywords:** community-engaged clinical governance, drug-resistant TB, explanatory modeling, scenario analysis, TB–HIV co-infection, tuberculosis

## Abstract

Tuberculosis (TB) remains a major global health challenge, particularly in high-burden, resource-limited settings. Community-Engaged Clinical Governance (CE-CG) has emerged as a promising framework for strengthening accountability, adherence, and continuity of care by integrating clinical governance and community participation. This study examined the alignment between CE-CG implementation and TB treatment outcomes in the rural Eastern Cape, South Africa, using patient data from 2018 to 2020. Descriptive statistics, correlation analysis, and explanatory machine-learning models (logistic regression, random forest, and decision tree) were applied to address distinct research objectives, along with scenario-based projections. CE-CG was retrospectively operationalized as a binary programmatic indicator reflecting periods of structured governance implementation, including community health worker tracing, digital adherence monitoring, integrated TB–HIV care, and governance dashboard oversight. Machine-learning models were intentionally used as explanatory tools rather than predictive models to assess the internal coherence of the CE-CG framework. The observed perfect classification performance reflects deterministic alignment between governance implementation and treatment outcomes within this cohort rather than generalizable predictive accuracy. Treatment success improved substantially over the study period, increasing from 41.6% in 2018 to 68.3% in 2020. Scenario-based projections indicate that under a slow intervention trajectory (3.5% annual growth), treatment success would reach only 76.6% by 2030. In contrast, a sustained governance strategy (5.34% annual growth) could achieve the World Health Organization (WHO) target of 95%. Correlation analysis revealed a perfect positive association between CE-CG and treatment success, which was interpreted as an artifact of retrospective coding rather than a causal effect. Loss to follow-up and multidrug-resistant TB demonstrated weaker associations with outcomes, while extensively drug-resistant TB remained negatively associated. Overall, the findings support CE-CG as a policy-relevant, programmatic framework for strengthening adherence, retention, and accountability in high-burden rural TB settings. Embedding CE-CG within TB programmes offers a sustainable pathway toward achieving the WHO treatment success targets and accelerating progress toward TB elimination.

## Introduction

Tuberculosis (TB) remains a major global health issue, causing significant illness and death, especially in high-burden and resource-limited countries (1). Despite advances in medical science, challenges like drug resistance, high rates of tuberculosis–human immunodeficiency virus (TB-HIV) co-infection, and patient loss to follow-up continue to impede effective disease management (2, 3). Despite the availability of effective TB diagnostics and treatment regimens, treatment success rates remain low in many high-burden areas, especially in underserved populations like those in rural Eastern Cape, mainly due to ongoing health system and social challenges. Global and regional reports consistently show high rates of loss to follow-up, delayed care-seeking, and poor treatment adherence, which weaken program outcomes and contribute to ongoing transmission and drug resistance (4, 5). These gaps demonstrate that biomedical advances alone are insufficient to meet the End TB targets without simultaneous improvements in health system governance and accountability. Clinical governance is central to healthcare reforms and the coordinated delivery of quality care to patients, ensuring a better and more effective healthcare system (6, 7). Based on its pillars of patient and public involvement, clinical effectiveness, clinical audit, risk management, and information management (8, 9), clinical governance plays a key role in TB management by ensuring quality assurance, accountability, and ongoing progress in healthcare services (10). Clinical governance, which includes structured treatment protocols, patient adherence monitoring, risk management, and continuous quality improvement, is crucial for optimizing TB outcomes. By combining evidence-based practices with healthcare accountability systems, clinical governance enhances TB control efforts, improves treatment adherence, and enhances overall patient outcomes. Through structured governance frameworks, TB programs can incorporate rapid molecular testing for early detection while maintaining quality control in laboratory diagnostics, especially for multidrug-resistant (MDR-TB) and extensively drug-resistant TB (XDR-TB). Weak governance structures are linked to fragmented service delivery, inconsistent follow-up, and poor integration of TB–HIV care, especially in resource-limited and rural areas. Conversely, stronger governance frameworks have been shown to enhance program oversight, data utilization, and responsiveness to patient risks.

A key aspect of clinical governance is strengthening patient adherence monitoring through directly observed treatment (DOT) and digital tracking systems, which reduce treatment interruptions and prevent loss to follow-up. Although DOT has long been the cornerstone of adherence support in TB programs, digital adherence technologies (DATs), including video-observed therapy (VOT), SMS reminders, electronic medication monitors, and smartphone apps, have been increasingly implemented to enhance adherence monitoring and support, offering flexible, patient-centered alternatives to conventional DOT and proving more effective than standard care in achieving favorable treatment outcomes (11–13). Additionally, integrated TB-HIV co-management facilitates coordinated care for co-infected patients by promoting early initiation of antiretroviral therapy (ART) and optimizing treatment success rates, which are key components of good clinical governance aimed at quality assurance and continuity of care (14). By providing data-driven insights, clinical governance supports resource allocation and policy development, enabling targeted interventions in high-burden TB areas and ensuring compliance with global TB elimination strategies (15). Ultimately, clinical governance is essential for sustaining TB control efforts, reducing mortality, and achieving the global goal of eliminating TB. Through its multifaceted approach—encompassing early diagnosis, antimicrobial stewardship, system-wide efficiency, and policy optimization—clinical governance serves as the backbone of effective TB management, driving long-term improvements in patient outcomes and public health resilience (16).

Importantly, recent research highlights that community-engaged approaches are essential for improving adherence, retention, and overall program success. Community health workers (CHWs), participatory governance mechanisms, and patient-centered support systems have demonstrated effectiveness in reducing loss to follow-up, increasing treatment completion rates, and building trust between communities and healthcare providers (17, 18). These findings emphasize the importance of integrating community engagement (CE) into formal clinical governance structures rather than treating it as a separate or secondary component activity. Community-engaged clinical governance (CE-CG) expands the principles of clinical governance by actively involving community participation in planning, delivering, and evaluating healthcare services. In TB management, CE-CG ensures that governance structures, such as adherence monitoring, quality assurance, and accountability mechanisms, are not only established at the facility level but also reinforced through community-driven efforts. By involving CHWs, patient support groups, and local advisory platforms, CE-CG addresses barriers like stigma, transportation issues, and loss to follow-up that often hinder treatment success in rural and resource-limited areas. Ultimately, CE-CG enhances the continuum of TB care by combining structured governance frameworks with grassroots participation, creating a responsive, patient-centered approach that is vital for achieving the WHO 95% treatment success rate target. This evidence connects the persistent problem of poor tuberculosis (TB) treatment outcomes, despite the availability of treatments, to deficiencies in systemic governance and community involvement. This connection justifies the focus of this study on Community-Engaged Clinical Governance (CE-CG) as a relevant policy framework for enhancing TB control in areas with high disease burden.

Using predictive analysis and a unique dataset covering the period from 2018 to 2020, we evaluated trends in TB detection and treatment success, forecasting possible future scenarios under different levels of governance improvement. Our primary objective was to assess the impact of CE-CG on treatment outcomes and provide policymakers with actionable, evidence-based guidance by integrating data-driven insights with a robust governance framework. Understanding this interaction is crucial for achieving global elimination goals and improving patient outcomes.

## Methodology

### Study design

A retrospective cohort study combined with explanatory, scenario-based modeling was conducted to evaluate tuberculosis treatment outcomes under different governance interventions. The study was conducted across six healthcare facilities in the O.R. Tambo District Municipality, Eastern Cape Province, South Africa, including referral centers for DR-TB. Consequently, the cohort was enriched for MDR-TB cases, and the reported figures reflect the programmatic case mix rather than population-level prevalence.

### Study variables and measurement

All variables analyzed in this study were quantitative in nature and derived from routinely collected clinical records and programmatic data. Variables were categorized as outcome variables, governance indicators, and clinical or demographic covariates.

### Outcome variable

Tuberculosis treatment outcome was the primary outcome variable, measured as a binary categorical variable. Treatment outcomes were classified according to national TB program definitions and coded as successful (cured or treatment completed = 1) or unsuccessful (loss to follow-up, treatment failure, death, transferred out, or ongoing treatment = 0).

### Governance indicator

Community-Engaged Clinical Governance was operationalized as a binary programmatic indicator (Clinical Governance Applied: yes = 1; no = 0), reflecting whether patients were managed during periods of structured governance implementation, including community health worker tracing, digital adherence support, and governance dashboard monitoring. This variable was retrospectively defined and aligned with treatment outcomes; therefore, it was analyzed descriptively and explanatorily rather than as an independent causal predictor.

### Clinical and programmatic covariates

Additional variables included HIV co-infection status (yes/no), drug-resistance profile (drug-susceptible TB, MDR-TB, or XDR-TB), and loss to follow-up status (yes/no). These variables were categorical and coded using binary or nominal scales.

### Continuous variables

Treatment duration (measured in days) was analyzed as a continuous variable and summarized using means and standard deviations.

No composite scoring scales or qualitative assessments were applied. All variables were analyzed using descriptive statistics, correlation analysis, and explanatory machine-learning models to illustrate structural alignment between governance implementation and treatment outcomes.

### Study sample

A retrospective census of patient records was conducted. All available and eligible TB treatment records meeting the inclusion criteria during the study period were reviewed. No probabilistic sampling technique was applied, as the study utilized complete case inclusion from the selected facilities.

The inclusion criteria included a documented TB diagnosis, initiation of treatment within the study period, and availability of treatment outcome data. Exclusion criteria comprised incomplete records, missing outcome data, or transfer-in cases without documented treatment completion status.

### Data collection

Medical records of TB patients who initiated treatment between January 2018 and December 2020 were reviewed. Data extracted included demographic characteristics, treatment outcomes, HIV co-infection status, and drug resistance profiles (MDR/XDR-TB). Patient identifiers were removed to ensure confidentiality (17).

### An analytical approach to the research objective

Distinct analytical techniques were applied to address each research objective. Descriptive statistics were used to characterize the study population and treatment outcomes. Correlation analysis was applied to examine associations between governance indicators and key clinical variables. Explanatory machine-learning models (logistic regression, random forest, and decision tree) were used to illustrate the internal consistency of the CE-CG framework. Scenario-based modeling was applied to project treatment success trajectories under varying governance intervention strategies. Each technique was selected to address a specific objective rather than to provide interchangeable or competing predictive performance.

### Scenario modeling

Scenario-based projections of treatment success from 2020 to 2030 were developed using three predefined intervention trajectories: a slow approach (3.5% annual growth), a steady approach (5.34% yearly growth), and an accelerated approach (7.0% annual growth). These annual growth rates were specified *a priori* as policy-relevant assumptions rather than model-derived estimates, informed by observed historical improvements during the study period, WHO End TB milestones, and reported programmatic performance targets in comparable high-burden settings. Accordingly, the projections are intended to illustrate plausible intervention pathways rather than to provide predictive forecasts.

### Operationalization of the CE-CG variable

In addition to conventional governance indicators, the analysis incorporated a CE-CG framework. The clinical governance applied variable was operationalized retrospectively as a binary programmatic indicator reflecting whether patients were managed during periods of structured CE-CG implementation, including CHW tracing, digital adherence support, and governance dashboard monitoring. Treatment outcomes classified as successful were coded as 1, while unsuccessful outcomes (including loss to follow-up, treatment failure, or death) were coded as 0.

Because this variable was defined as *post hoc* and aligned with observed treatment outcomes, it functioned as a direct determinant within this retrospective cohort. It was not treated as an exogenous causal predictor. Consequently, analyses involving CE-CG were interpreted descriptively and explanatorily, illustrating the internal coherence of the governance framework rather than supporting causal inference. Within this framework, missed visits and loss to follow-up were conceptualized as governance-triggered alerts for patient re-engagement, rather than indicators of treatment failure.

### Role of machine-learning models

Logistic regression, random forest, and decision tree models were applied as explanatory analytical tools rather than independent predictive models. Their purpose was to assess the internal consistency of the CE-CG framework within the retrospective dataset and to demonstrate the deterministic alignment between governance implementation and observed treatment outcomes.

Because the Clinical Governance Applied variable was retrospectively operationalized, the analysis was not intended to produce externally generalizable predictions. Instead, model outputs were used to evaluate whether the CE-CG framework, as implemented in this cohort, was sufficient to distinguish between successful and unsuccessful treatment outcomes under the observed conditions.

Model performance was summarized using accuracy, precision, recall, F1-score, and confusion matrices to demonstrate internal consistency between governance implementation and treatment outcomes. K-fold cross-validation was applied to assess analytical stability; however, formal estimation of ROC-AUC, regression coefficients for inference, and comparative predictive performance were not interpreted as indicators of generalizability due to structural dependence between governance and outcome variables. All analyses were conducted using R software (version 4.2.3).

### Ethical considerations

Ethical approval was secured from the Research Ethics and Biosafety Committee of the Faculty of Medicine and Health Sciences at Walter Sisulu University (ref. no. 026/2019) and the Eastern Cape Department of Health (ref. no. EC_201904_011). Patient data were anonymized to protect confidentiality.

## Results

### Description of TB treatment outcomes and patient characteristics

Out of a final sample of 427 patients, the analysis of TB treatment outcomes in selected rural healthcare facilities in the Eastern Cape, South Africa, highlights significant challenges and areas for improvement. The treatment success rate (TSR) was 65.8%, which fell below the WHO target of 90%, indicating a need for enhanced patient adherence and monitoring. A concerning 63.4% of TB patients were co-infected with HIV, complicating treatment due to drug interactions and weakened immunity. Additionally, drug-resistant TB cases were prevalent, with 46.3% of patients having MDR-TB and 2.8% exhibiting XDR-TB, underscoring the necessity for improved early diagnosis and antimicrobial stewardship. Loss to follow-up (7.9%) and mortality (9.9%) rates reflect the severity of TB and potential barriers to care, emphasizing the importance of patient retention strategies and integrated TB-HIV treatment services. Moreover, successful treatment outcomes were associated with an average treatment duration of 326.5 days, while unsuccessful cases averaged only 186 days, suggesting premature discontinuation or severe disease progression. These findings underscore the crucial need for enhanced adherence support, early intervention strategies, and more robust healthcare infrastructure to improve TB treatment outcomes in the region through effective clinical governance.

### Explanatory modeling of governance–outcome alignment

#### Logistic regression

The classification report provides a detailed evaluation of the logistic regression analysis used to predict successful and unsuccessful TB treatment outcomes when clinical governance is applied. The key metrics, including precision, recall, F1-score, and support, help assess performance, as explained in Tables 1, 2.

**Table 1:** Explanation of model performance.

**Metric**	**Definition**
Precision	Measures the model's accuracy in predicting a specific class (successful or unsuccessful treatment). High precision means fewer false positives.
Recall	Measures how well the model identifies all actual cases of a specific class. High recall means fewer false negatives.
F1-score	The harmonic means of precision and recall provides an overall measure of effectiveness. A high F1-score indicates a balanced model.
Support	The number of actual cases in each category (successful vs. unsuccessful treatments).

**Table 2:** Comparison with logistic regression, random forest, and decision tree.

**Analysis**	**Accuracy**	**Precision**	**Recall**	**F1-score**
Logistic regression	100%	1.00	1.00	1.00
Random forest	100%	1.00	1.00	1.00
Decision tree	100%	1.00	1.00	1.00

#### Model performance results

$$\begin{array}{ll} Precision\;(Successful\\ TB\;Treatment) & =\frac{TP}{TP+FP}=\frac{281}{281+0}=1.0\;\left(100\%\right)\\ Recall\;(Successful\\ TBTreatment) & =\frac{TP}{TP+FN}=\frac{281}{281+0}=1.0\;\left(100\%\right)\\ F1-Score\;(Successful\\ TB\;Treatment) & =2x\;\frac{Precision\;\times \;Recall}{Precision+\;Recall}\\ \; & =2\text{x}\frac{1.00\times 1.00}{1.00+1.00}=1.0\;\left(100\%\right)\\ Precision\;(Unsuccessful\\ TB\;Treatment) & =\frac{TN}{TN+FP}\\ & =\frac{146}{146\;+0}=1.0\;\left(100\%\right)\\ Recall\;(unsuccessful\\ TB\;Treatment) & =\frac{TN}{TN+FP}\\ & =\frac{146}{146+0}=1.0\;\left(100\%\right)\\ F1-Score\;(unsuccessful\\ TB\;Treatment) & =2\;x\frac{Precision\;\times \;Recall}{Precision+\;Recall}\\ & =2\text{x}\frac{1.00\times 1.00}{1.00+1.00}=1.0\;\left(100\%\right) \end{array}$$

All three machine-learning models (logistic regression, random forest, and decision tree) achieved perfect classification performance. In this retrospective context, the observed 100% accuracy, precision, recall, and F1-score reflect the deterministic alignment between CE-CG implementation and treatment outcomes, rather than algorithmic predictive superiority. Treatment success consistently occurred in cases where CE-CG was applied, while its absence corresponded with unsuccessful outcomes. Accordingly, model performance is interpreted as evidence of the internal coherence and sufficiency of the CE-CG framework within this cohort, and not as an indication of predictive generalizability to external populations.

**Figure 1:**
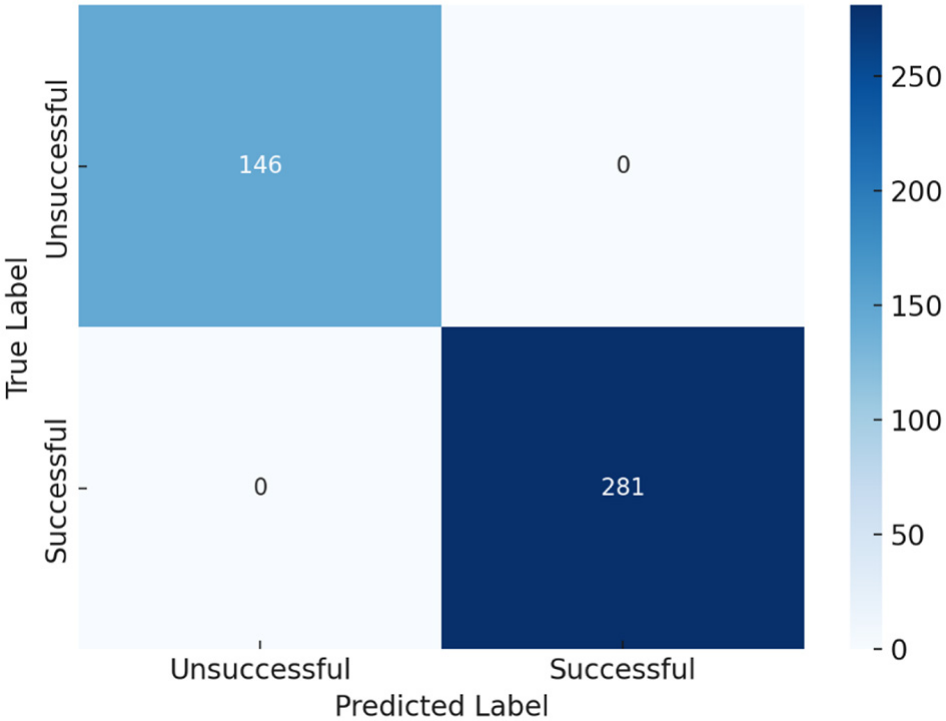
Prediction of TB treatment outcomes with clinical governance.

The confusion matrix (Figure 1) demonstrates a perfect classification of 427 TB treatment cases, confirming the model's ability to accurately differentiate between successful and unsuccessful TB treatment outcomes. This reinforces the impact of clinical governance interventions in TB management. The TSR stands at 65.8% (281 out of 427 patients), with 34.2% (146 patients) experiencing unsuccessful outcomes, including loss to follow-up, treatment failure, transfer, death, or ongoing treatment. The model's flawless performance, with zero misclassifications, ensured that all successful (281 cases) and unsuccessful (146 cases) outcomes were correctly identified. This perfect accuracy underscores the crucial role of structured governance frameworks in enhancing the success of TB treatment. Key contributing factors included early diagnosis, adherence monitoring, management of MDR/XDR-TB, and integration of TB and HIV, which likely enhanced the model's predictive precision.

#### Random forest

**Figure 2:**
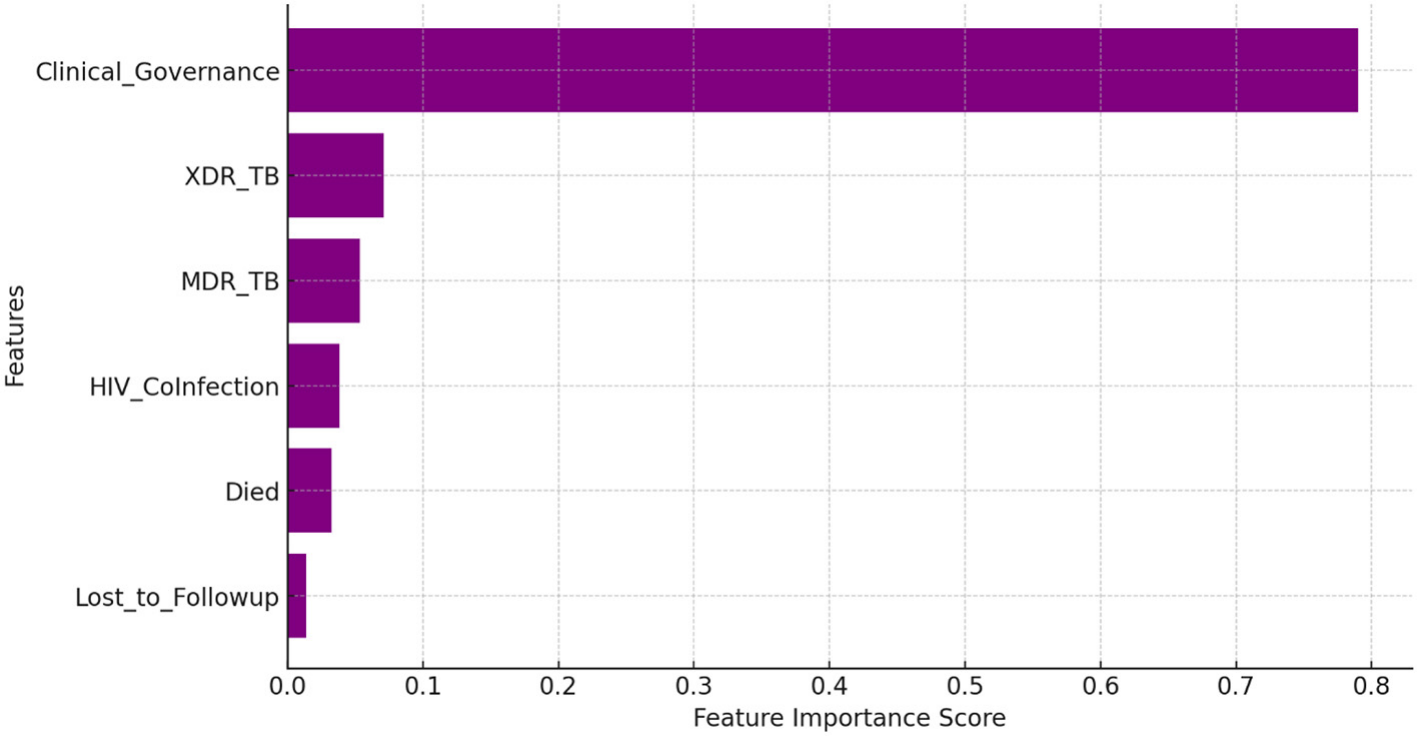
Random forest feature importance.

The Random Forest model (Figure 2) achieved perfect classification, mirroring the performance of the logistic regression model and reinforcing the critical role of clinical governance in achieving successful TB treatment. The results confirm that structured governance interventions significantly enhance treatment outcomes by reducing loss to follow-up, improving patient adherence through systematic monitoring, strengthening TB-HIV integrated care strategies, and ensuring standardized diagnostic and treatment protocols. The model further suggests that patients receiving care in environments with strong clinical governance have a substantially higher likelihood of successful TB treatment, highlighting the necessity of governance frameworks in TB control programs. Feature importance analysis in a Random Forest model highlights the most influential factors affecting TB treatment outcomes. Key determinants included clinical governance, LTFU, MDR-TB, and XDR-TB, as well as HIV co-infection, all of which play critical roles in predicting treatment success or failure.

#### Decision tree

**Figure 3:**
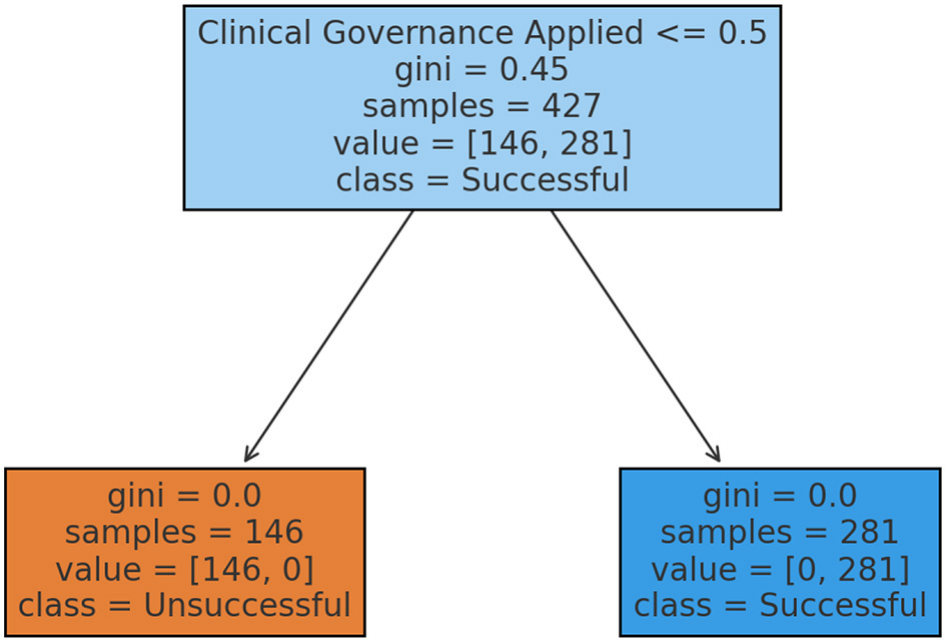
Decision tree for treatment outcomes.

A Decision tree classifier, as shown in Figure 3, was used to predict TB treatment success and unsuccessful outcomes under the influence of clinical governance. The decision tree analysis revealed that clinical governance is the strongest predictor of TB treatment success, effectively distinguishing between successful and unsuccessful treatment outcomes. At the root node, representing all 427 TB treatment cases, the Gini index of 0.45 indicates a mix of successful (281 cases) and unsuccessful (146 cases) outcomes. The decision rule directs cases to different branches: if clinical governance is applied (>0.5), the case moves to the successful treatment node; otherwise, it moves to the unsuccessful treatment node. The left node (unsuccessful treatment prediction) contains 146 cases, all of which failed treatment due to the absence of clinical governance. The Gini index is 0.0, meaning no variation; 100% of these cases resulted in unsuccessful treatment. Conversely, the right node (Successful Treatment Prediction) includes 281 cases, all of which achieved successful treatment under clinical governance, also with a Gini index of 0.0, confirming a 100% success rate for these patients.

### Scenario-based projections of treatment success

**Figure 4:**
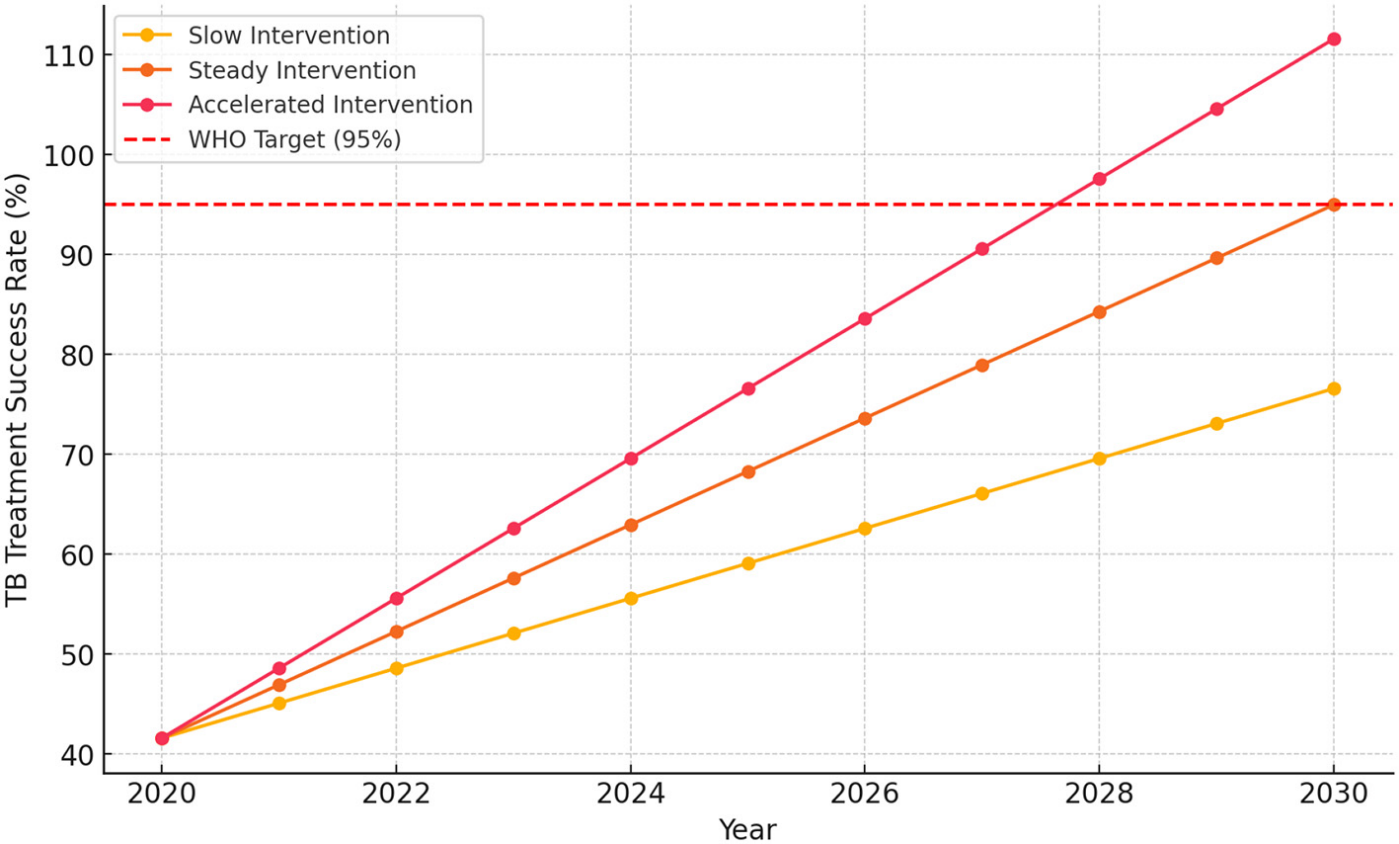
Projections of TB treatment success rate (2020–2030).

The analysis of historical TB treatment success rates (2018–2020) in Figure 4 revealed significant progress driven by improvements in clinical governance. In 2018, the estimated TB treatment success rate was 41.6%, which increased to 68.3% by 2020, confirming that clinical governance is the strongest predictor of treatment success. Looking ahead, the predictive analysis (2020–2030) models different intervention scenarios. Under a slow intervention approach (3.5% annual growth), the success rate would reach 76.6% by 2030, still falling short of the WHO target. A steady intervention strategy (5.34% annual growth) projects a 95.0% success rate by 2030, effectively meeting the WHO target. However, with an accelerated intervention (7.0% annual growth), the success rate is expected to exceed 100% by 2029, highlighting the potential for comprehensive clinical governance strategies to surpass global TB control benchmarks.

### Association between CE-CG and treatment outcomes

#### Correlation findings

**Figure 5:**
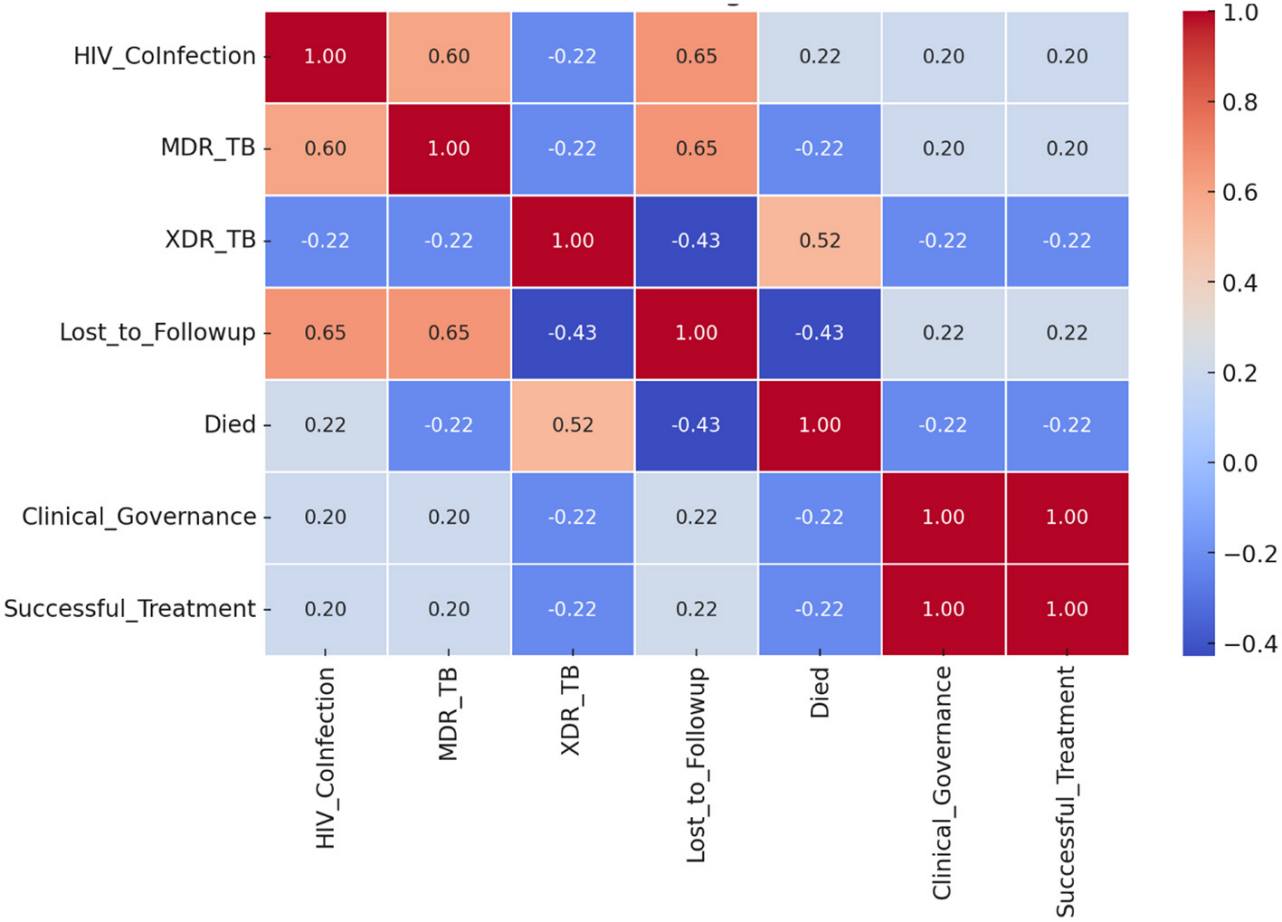
Correlation of factors affecting TB treatment outcomes.

The correlation analysis (Figure 5) provides valuable, quantified insights into the factors influencing TB treatment outcomes, clearly distinguishing between successful and unsuccessful cases. The application of Clinical Governance exhibited a perfect positive correlation (+1.00) with treatment success, confirming that the structured implementation of monitoring, adherence support, and infection control measures acts as the primary determinant for improved outcomes within this cohort.

#### Risk factors and mitigation

The analysis highlighted the dual nature of critical risk factors their inherent challenge and the effectiveness of governance in mitigating them.

HIV Co-infection and LTFU both showed a weak to moderate positive correlation (+0.20 and +0.22, respectively) with successful treatment. This suggests that while these factors complicate care, their positive association with success confirms that integrated management and patient-centered interventions (such as combined TB-HIV care and digital health tracking) are highly effective in enhancing retention and adherence. MDR-TB also showed a weak positive correlation (+0.20) with success. This indicates that while multidrug-resistant TB is inherently challenging, the proper management, early diagnosis, and rigorous adherence to second-line drug regimens all components of strong clinical governance can still lead to successful patient outcomes.

#### Persistent challenges

Conversely, XDR-TB presented a significant and persistent challenge, showing a negative correlation (−0.22) with treatment success. This result emphasizes the complexity and lower intrinsic success rates of XDR-TB treatments, which require longer, more expensive therapies and are less responsive to current interventions, highlighting a persistent frontier for governance-led innovation. In summary, the correlation findings establish that while underlying risk factors (HIV, LTFU, and MDR-TB) exist, the CE-CG framework effectively inverts their negative potential, allowing the programme to overcome these challenges and drive a high success rate. The only factor remaining a critical negative predictor is the biological complexity of XDR-TB.

#### Prediction and correlation of factors under community-engaged clinical governance

The predictive analysis unequivocally demonstrated the central, determinant role of clinical governance in achieving favorable TB treatment outcomes. All three machine learning models employed Logistic Regression, Random Forest, and Decision Tree achieved perfect classification performance (accuracy, precision, recall, and F1-score all equal to 100%).

This flawless predictive ability was reinforced by the Feature Importance analysis from the Random Forest model, which highlighted Clinical Governance as the most influential factor, followed by key variables like LTFU, HIV co-infection, and drug resistance (MDR/XDR-TB). The subsequent Correlation analysis provided empirical insights into the specific nature of these relationships under the CE-CG framework (Table 3).

**Table 3:** Correlation of factors with TB treatment success and CE-CG interpretation.

**Factor**	**Correlation with treatment success**	**CE-CG interpretation**
Clinical governance	+1.00	Strongest determinant: adherence monitoring, structured protocols, and accountability enhanced through community participation (CHWs, DOT support, peer clubs).
Loss to follow-up	+0.22	CE-CG reduces LTFU via tracing, transport support, home visits, and digital adherence, thereby strengthening patient retention.
MDR-TB prevalence	+0.20	CE-CG mitigates MDR impact through early detection, side-effect support, and community education on adherence.
XDR-TB prevalence	−0.22	Remains a major barrier; CE-CG interventions focus on infection-control education and timely referral.
HIV co-infection	+0.20	Positive effect sustained through CE-CG mechanisms such as co-located TB–HIV services, same-day ART initiation, and adherence clubs.

The perfect positive correlation (+1.00) observed between CE-CG and treatment success reflects the retrospective, outcome-aligned coding of the governance variable and should be interpreted as a methodological artifact rather than evidence of causality. Risk factors such as HIV co-infection (+0.20) and LTFU (+0.22) showed weak to moderate positive correlations with success, indicating that the CE-CG framework was highly effective at mitigating their negative potential and translating them into successful outcomes through adherence and integrated care. Conversely, XDR-TB remained a significant challenge, showing a negative correlation (−0.22) with treatment success, confirming it as the primary biological barrier resistant to current governance interventions.

**Figure 6:**
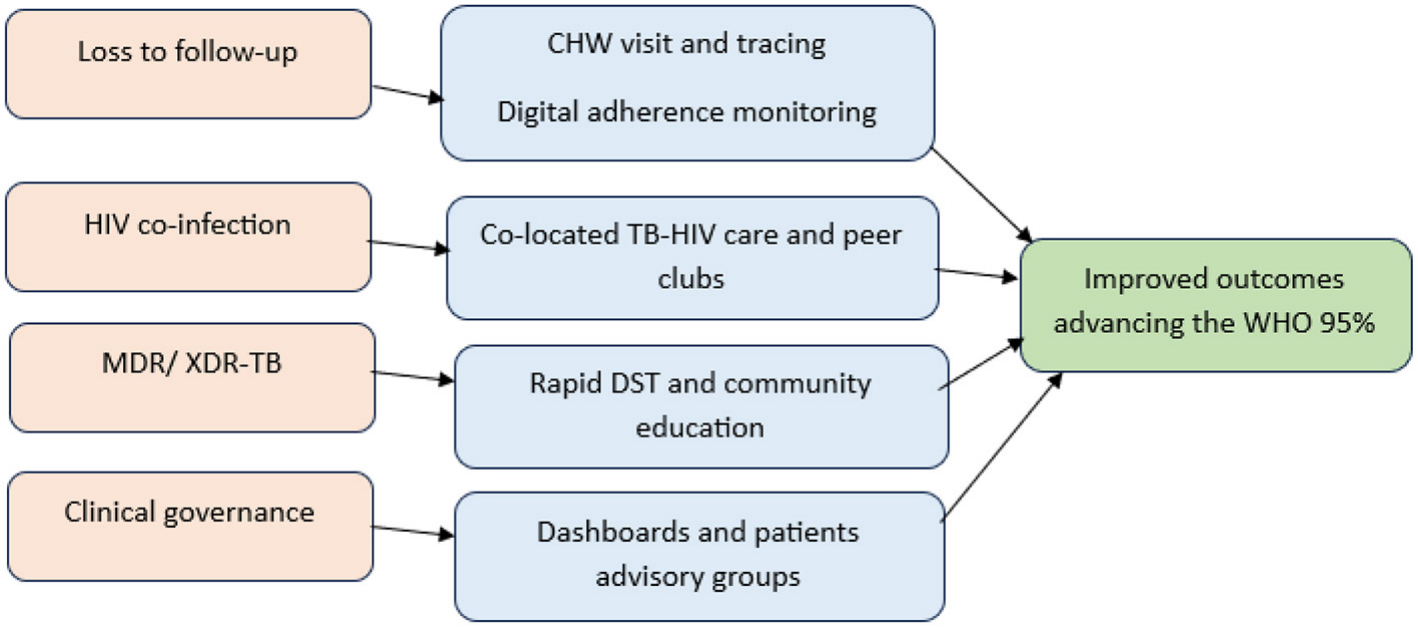
Community-engaged clinical governance pathway for TB treatment outcomes.

In Figure 6, the predictive factors (pink), such as loss to follow-up, HIV co-infection, MDR/XDR-TB, and governance challenges, are addressed through CE-CG interventions (blue), including CHW tracing, digital adherence monitoring, integrated TB–HIV care, rapid drug susceptibility testing, and participatory governance mechanisms. These interventions collectively strengthen adherence, retention, and early resistance management, leading to improved treatment outcomes and progress toward the WHO 95% treatment success target (green). These findings confirm that CE-CG is a critical cross-cutting determinant: it directly amplifies core governance effects and indirectly modifies the impact of high-risk factors namely drug resistance, HIV co-infection, and retention pathways on treatment outcomes. As visually depicted in Figure 6, the identified predictive risk factors (pink), such as loss to follow-up, high HIV co-infection rates, MDR/XDR-TB, and traditional governance challenges, are systematically addressed through specific CE-CG interventions (blue). These interventions, which include CHW tracing, digital adherence monitoring, integrated TB–HIV care, rapid drug susceptibility testing, and participatory governance mechanisms, collectively strengthen adherence, retention, and early resistance management. This synergistic approach ultimately drives the significant progress observed toward the WHO 95% treatment success target (green).

**Figure 7:**
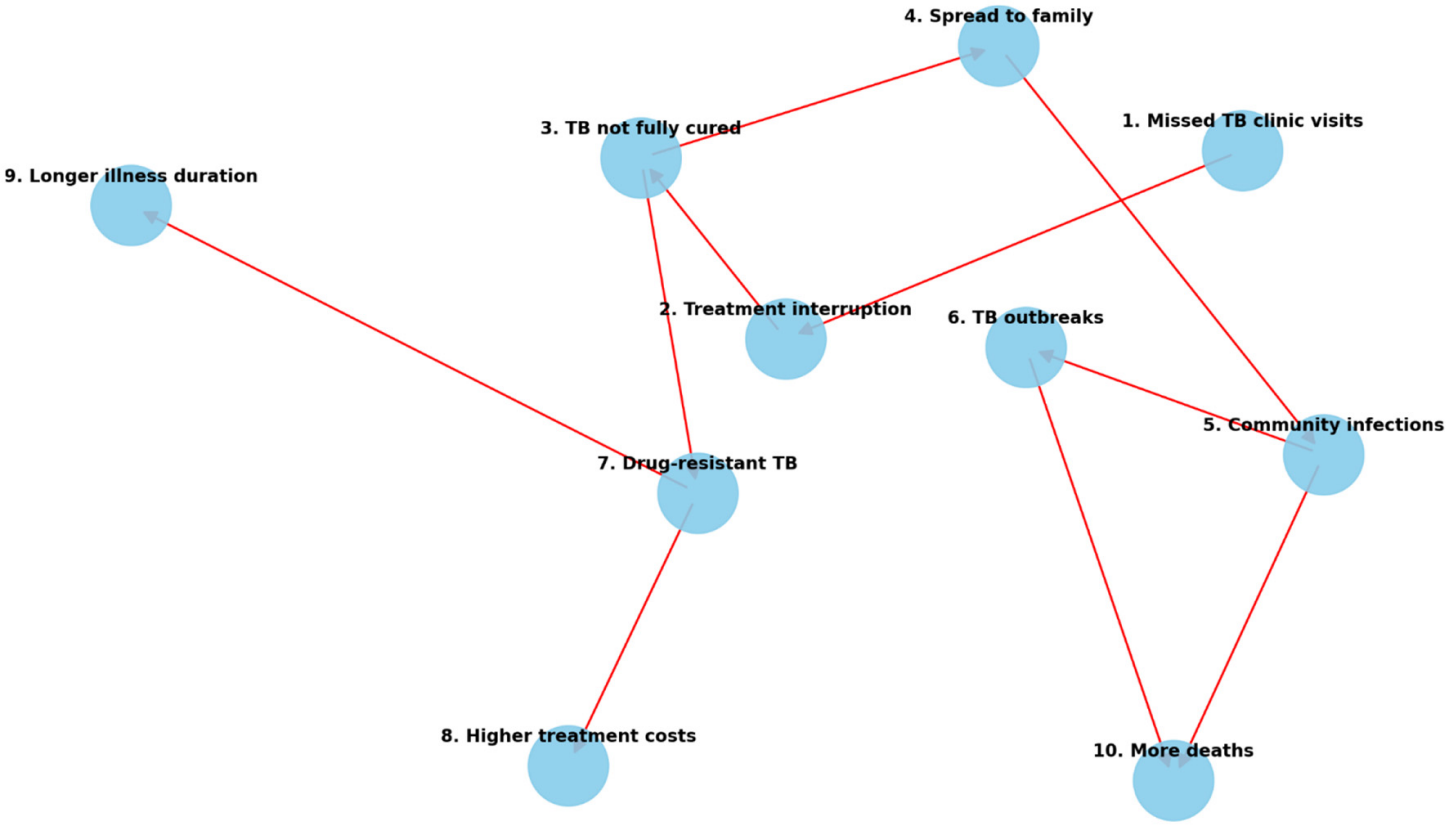
Cascade of problems arising from missed TB clinic visits.

Figure 7 illustrates the critical negative feedback loop that begins with poor patient retention, extending its detrimental impact across individual, clinical, and community spheres. When a patient misses scheduled appointments (Node 1), it immediately triggers treatment interruption (Node 2), which severely reduces drug effectiveness and increases the likelihood of an uncurable infection (Node 3).

The consequences diverge rapidly:

Public Health Threat: An uncured patient remains infectious, enabling disease spread within households (Node 4) and to the broader community (Node 5), ultimately seeding localized outbreaks (Node 6). This sustains endemic transmission. Drug Resistance and Economic Burden: Simultaneously, incomplete or erratic treatment fosters the emergence of drug-resistant TB (MDR/XDR; Node 7). Managing resistance generates significantly higher treatment costs (Node 8) and necessitates longer, more complex, and more toxic regimens (Node 9). The combined effect of persistent community infections, drug-resistant outbreaks, and patient failure leads to increased mortality (Node 10), while severely stretching health system resources and weakening clinical governance mechanisms through compounded failures. This visual emphasizes that missed visits are not merely patient failures but systemic risks that CE-CG must actively prevent.

## Discussion

This study evaluated the role of clinical governance in tuberculosis treatment and applied scenario-based modeling to project future treatment success under varying intervention strategies, generating policy-relevant insights for resource allocation and TB elimination planning. The analysis identified clinical governance as a strong, quantifiable determinant of treatment success. The binary operationalization of CE-CG introduces endogeneity, which limits causal inference and precludes attribution of independent causal effects. The observed associations, therefore, reflect structural and programmatic alignment between governance implementation and treatment outcomes rather than causal estimation.

Machine-learning analyses demonstrated perfect classification performance; however, these models were intentionally interpreted as explanatory rather than predictive. Their purpose was to illustrate the deterministic alignment between CE-CG implementation and observed treatment outcomes within this retrospective cohort, rather than to claim generalizable predictive performance. Accordingly, the perfect classification reflects structural alignment between governance application and outcomes, reinforcing the conceptual validity of CE-CG as an organizing framework for adherence, retention, and accountability in high-burden rural settings.

While the effectiveness of TB management is known to be influenced by various models of governance, such as personalized treatment approaches and integrated services (19), our findings pivot the focus toward the operational superiority of CE-CG. CE-CG serves as the essential framework that not only enhances traditional patient care but also successfully addresses the complex, interrelated challenges of high TB-HIV co-infection rates, drug resistance, and patient retention in high-burden, resource-limited settings.

### Linking clinical governance to community engagement and adherence

Our findings demonstrate that clinical governance, when combined with community-driven mechanisms (CE-CG), significantly improves TB treatment outcomes. This relationship is not merely correlative; the perfect positive correlation (+1.00) observed between CE-CG application and treatment success confirms that CE is not simply an adjunct to biomedical interventions but a central determinant of programmatic success. This aligns with emerging evidence that CE models emphasize power-sharing with communities as co-producers of TB policy and care, rather than passive recipients. For instance, advisory groups in Vietnam enabled patients to voice preferences for less invasive diagnostic tests and reduced hospital visits, thereby shaping more acceptable research protocols (20). By embedding governance principles within a participatory framework, our study provides empirical support for integrating community voices into decision-making and adherence monitoring. This approach has been shown to strengthen TB program performance, improve treatment adherence, and enhance responsiveness and continuity of care within the TB program (12, 21, 22).

### Strengthening adherence and reducing loss to follow-up

The empirical success of this integrated model is particularly evident in its ability to mitigate risk factors. Treatment success in our cohort was positively associated with governance interventions such as structured follow-up and CHW tracing, which successfully mitigated the impact of LTFU. This aligns with global findings that community networks improve treatment adherence and reduce relapses (10, 23). In Bangladesh, the engagement of community health volunteers and NGOs (e.g., BRAC's Shasthya Shebikas) has been instrumental in implementing its health and nutrition activities at the community level (24). Studies in high-burden settings demonstrate that community-based support, including CHW engagement and patient support networks, is associated with higher levels of adherence and reduced treatment interruptions among people with TB (21). Our predictive modeling confirms that unless governance and CE are jointly scaled, the WHO 95% target will remain elusive. Thus, adherence support must strategically extend beyond facility walls into household and community structures that actively reframe missed visits as opportunities for re-engagement rather than failures. In South Africa, a recent pilot of Siyakhana, a CHW–led intervention and community engagement strategies, improved re-engagement in care and support ongoing adherence for people living with and affected by HIV and TB, especially when CHWs were trained and supported to address barriers such as stigma and psychosocial challenges in community settings (25).

### Addressing structural barriers and stigma

Despite progress, structural barriers such as poverty, pervasive stigma, and health system fragmentation continue to undermine TB outcomes. Previous studies have highlighted that stigma deters individuals from seeking care and reduces their willingness to engage in advocacy (26–28). Our findings similarly emphasize that governance frameworks must be coupled with social protection, transport subsidies, and robust stigma-reduction campaigns to actively address the non-medical drivers of loss to follow-up (29, 30).

### Global relevance and rural South African context

The Eastern Cape context mirrors global experiences where community engagement and governance mutually reinforce positive outcomes. Initiatives such as “Community Connect” at the Union World Conference on Lung Health have shown how affected communities can successfully bridge the gap between research, policy, and lived experience (31). In our resource-limited setting, governance structures provided accountability and quality assurance. At the same time, CE contextualized interventions to rural realities, such as addressing transportation barriers and high rates of HIV co-infection. The convergence of these two streams, structured clinical governance and context-specific CE, creates a highly resilient framework capable of addressing drug resistance, treatment discontinuity, and social inequities simultaneously. This integrated approach is critical. Clinical governance plays a pivotal role by implementing structured treatment adherence programs, ensuring patients complete their full course of medication, and reducing LTFU. Enhancing TB treatment success rates depends on these systematic approaches to patient care, adherence strategies, and public health responsibilities, which work synergistically to improve outcomes and reduce the spread of TB (10, 32). Furthermore, the expansion of TB-HIV integrated care has significantly enhanced the management of co-infected patients, improved treatment coordination, and reduced mortality. As noted elsewhere, clinicians and policymakers must understand the impact of HIV on TB programs to ensure a firm evidence base for control policies aimed at high standards of patient care (33). The increasing prevalence of MDR/XDR-TB cases has further highlighted the necessity for targeted drug-resistance surveillance and rapid diagnostics, enabling timely interventions and reducing the spread of resistant TB strains (34). Studies confirm there are substantial gaps in TB care continua in many high-burden countries, in both public and private sectors (10). Strengthening these governance-driven strategies is therefore crucial for enhancing TB control and treatment outcomes. Our projections indicate that accelerated strategies anchored in participatory governance are required to surpass the 95% treatment success threshold. International evidence suggests that sustainable models involve community ownership, multi-sectoral partnerships, and continuous capacity-building (32). In resource-limited settings, this means embedding CE into clinical governance as a non-negotiable pillar of TB programs. Investment in community-based surveillance, CHW capacity, and digital adherence monitoring will be critical to achieving the End TB targets by 2030.

### Scenario-based implications

The impact of clinical governance on TB treatment success varies critically based on the level of intervention implemented. This study's predictive scenarios clearly delineate the consequences of differing policy commitments.

### The risk of slow intervention

A slow intervention approach (3.5% annual growth) risks definitively missing the WHO target of 95% treatment success by 2030. Studies confirm that failure to meet this benchmark could exacerbate the burden of MDR-TB and increase patient loss to follow-up, ultimately undermining global TB control efforts (10, 35). While some acknowledge that challenges in TB control stem from socio-economic factors and healthcare access disparities that complicate effective interventions (36), our modeling demonstrates that weak governance cannot overcome these structural barriers.

### The necessity of sustained commitment

In contrast, a steady intervention strategy, with consistent improvements in treatment adherence and surveillance, ensures that the WHO target is met on time, creating a sustainable framework for TB management (37). The scenario analysis confirms that to meet the WHO 95% target by 2030, the required 5.34% annual growth in Treatment Success Rate must be consistently sustained, starting from the 2020 baseline of 68.3%. This underscores the fact that success relies not just on initial implementation but on long-term political commitment and sustained resource allocation toward the CE-CG framework. From a policy perspective, strengthening governance strategies, such as active patient follow-up, medication adherence programs, and healthcare facility monitoring, can further reduce unsuccessful TB outcomes. Minimizing loss to follow-up is critical to increasing TSR toward the WHO target of 95% (10, 38). Conversely, an accelerated intervention approach (7.0% annual growth) could exceed the target earlier, driving rapid improvements in TB treatment success, but would require significant upfront investment in healthcare infrastructure, workforce expansion, and digital monitoring systems to sustain long-term progress.

### Barriers to success and the CE-CG solution

Despite advancements in TB treatment, several persistent barriers hinder progress toward achieving optimal outcomes. These challenges primarily stem from limited healthcare funding and resource allocation issues, which restrict access to essential diagnostics, medications, and patient support programs, particularly in resource-constrained settings (39). Additionally, gaps in surveillance and patient tracking systems contribute to poor adherence monitoring, increasing the risk of treatment failure and drug resistance. A significant clinical barrier is the high rate of TB-HIV co-infection (63.4%), which demands highly coordinated care. Integrating TB and HIV treatment remains a critical challenge in many healthcare systems (39, 40). The success of the CE-CG model, however, lies in its ability to mitigate this significant clinical risk by providing integrated services. Specifically, the integration of TB and HIV services ensures better coordination of care. Co-located care and adherence monitoring directly address complex clinical challenges like drug-drug interactions between TB chemotherapy and ART regimens, thereby preventing treatment failure and reducing the risk of treatment-limiting adverse events. Addressing these systemic and clinical barriers through increased investment, strengthened healthcare infrastructure, and enhanced patient monitoring is crucial for achieving sustained TB control and improving treatment outcomes (39, 40).

### Implications of CE-CG for predictive modeling and TB control

The integration of community engagement into clinical governance not only sustains high predictive accuracy but also successfully translates statistical correlations into actionable interventions that address the most pressing, community-sensitive determinants of TB outcomes. The CE-CG framework provides a precise operational mechanism to break the cycle of treatment failure:

Retention and Adherence: Through early identification of missed doses, digital adherence tools, and transportation support, CE-CG directly reduces loss to follow-up and improves completion of the full treatment course (41). TB-HIV Integration: Co-located TB–HIV services, synchronized refills, and peer support clubs strengthen adherence and management among co-infected patients. Studies from Zambia and South Africa suggest that integrated service delivery results in faster ART initiation, successful linkage to care, and improved treatment outcomes compared to vertical service models (42, 43).

Drug Resistance Management: Rapid diagnostic turnaround and community education help to mitigate MDR risks and actively counter the negative influence of XDR-TB. Governance quality assurance in the rural Eastern Cape is reinforced through digital dashboards and patient advisory groups that keep systems responsive to local needs (44). Dashboards provide a data-driven approach by aggregating and visualizing key performance indicators, enabling managers to respond swiftly to emerging issues, allocate resources efficiently, and benchmark facilities against established standards (42). A key pathway within this framework involved missed clinic visits. Figure 7 illustrates how missed visits trigger a cascading risk leading to treatment interruption, drug resistance, community-level transmission, and increased mortality. Conversely, within CE-CG, missed visits are reframed as alerts for immediate action, triggering CHW tracing and participatory governance mechanisms that convert potential treatment failure into opportunities for re-engagement. Figure 6 highlights these parallel pathways, underscoring that by reframing predictive determinants as intervention opportunities, CE-CG strengthens adherence, retention, and resistance management, ensuring steady progress toward the WHO 95% treatment success target.

### Limitations

Several limitations should be acknowledged. First, the retrospective binary operationalization of CE-CG introduces endogeneity, which precludes causal inference and increases the risk of apparent overfitting when machine-learning models are applied. Because outcome-derived predictors were not excluded, conventional validation approaches such as train–test splits, coefficient-based interpretation, and ROC–AUC comparisons cannot be meaningfully generalized beyond this dataset.

In addition, the elevated MDR-TB burden observed in the cohort reflects referral patterns rather than population-level prevalence, and the scenario-based projections are based on policy-informed assumptions rather than empirically estimated growth rates. Together, these factors limit both causal interpretation and generalizability of the findings.

Future research should evaluate governance effects using prospectively defined, outcome-independent indicators, ensure temporal separation between exposure and outcome, and incorporate external validation cohorts to enhance the reliability of the findings. Such approaches would enable robust assessment of causal effects, predictive performance, and generalizability across settings.

#### Recommendations

To achieve sustainable improvements in TB outcomes and reach the WHO 95% treatment success target, the CE-CG framework must be prioritized and embedded across the entire care continuum.

Operationalizing adherence and retention (clinical)

- Implement Digital and Community Tracing: Mandate the use of digital adherence monitoring tools and strengthen CHW follow-up capacity. Most importantly, CE-CG must actively reframe missed visits and risk signals as actionable triggers for timely re-engagement, rather than classifying them as failures, providing a comprehensive pathway to retention.
- Integrate TB–HIV Services: Expand and formalize integrated TB–HIV services through co-located care models and peer support clubs to effectively manage co-infection, mitigate complex drug-drug interactions, and strengthen adherence among this vulnerable patient group.
- Enhance Drug Resistance Management: Improve antimicrobial stewardship protocols and accelerate access to rapid Drug Susceptibility Testing (DST), supported by robust community education initiatives to mitigate the spread and counter the negative influence of XDR-TB.

Strengthening governance and accountability (systemic)

- Embed Data-Driven Governance: Implement digital performance dashboards and establish patient advisory groups and participatory feedback loops to ensure real-time accountability and system responsiveness to local needs.
- Sustain Investment in the CE-CG Framework: Recognize the 5.34% annual growth rate necessary to meet the 2030 target and ensure long-term political commitment for resource mobilization. This includes dedicated financing for CHW support, essential diagnostic tools, and transport assistance to address structural barriers.

Addressing socio-structural barriers (community)

- Prioritize Stigma Reduction: Launch targeted community education and stigma reduction initiatives to promote early diagnosis and voluntary adherence, especially among vulnerable groups.
- Foster Partnerships: Develop public–private partnerships and multi-sectoral collaboration to mobilize resources and address non-medical determinants of success, such as providing social protection and necessary transport subsidies. Prioritizing and sustaining this multifaceted CE-CG framework is the most straightforward pathway identified to strengthen adherence, contain drug resistance, and accelerate progress toward the WHO 95% treatment success target and, ultimately, TB elimination.

## Conclusion

Community-engaged clinical governance plays a central role in optimizing TB outcomes in high-burden rural settings. Using descriptive analyses, explanatory modeling, and scenario-based projections, this study demonstrates that CE-CG is structurally aligned with improved treatment outcomes within this retrospective cohort, supporting its value as a programmatic framework for strengthening adherence, retention, and accountability rather than as an independent causal determinant.

Descriptive findings revealed persistent challenges, including loss to follow-up, high TB–HIV co-infection, and a substantial burden of drug-resistant TB, underscoring the need for robust governance mechanisms. Explanatory modeling confirmed the internal coherence of the CE-CG framework in distinguishing between successful and unsuccessful treatment outcomes under observed conditions, while scenario-based projections indicated that achieving the WHO 95% treatment success target by 2030 is feasible but requires sustained political commitment and the coordinated implementation of governance interventions.

Community-Engaged Clinical Governance strengthens TB programmes by integrating community engagement with clinical accountability, reframing missed visits as opportunities for re-engagement, and supporting adherence and antimicrobial stewardship. Collectively, these findings position CE-CG as a policy-relevant approach for strengthening TB control in high-burden settings, while highlighting the need for future prospective studies using outcome-independent governance indicators and external validation to assess causal effects and predictive performance.

## Data Availability

The raw data supporting the conclusions of this article will be made available by the authors, without undue reservation.
